# Impact of Adjuvant Chemotherapy on Survival Outcomes in Intermediate‐ and High‐Risk ER+/HER2− Breast Cancer Stratified by Genomic Profiling: A Meta‐Analysis

**DOI:** 10.1155/ijbc/4973135

**Published:** 2026-04-06

**Authors:** Max Seabrook, Ahamed S. M. Navas, Hannah-Maria Francis, Zina Aladili, Ahsan Rao

**Affiliations:** ^1^ General Surgery, Royal Cornwall Hospitals NHS Trust, Truro, UK, rcht.nhs.uk; ^2^ Department of Surgery, Mid and South Essex NHS Foundation Trust, Basildon, UK

## Abstract

**Background:**

Genomic recurrence score (RS) testing guides adjuvant treatment decisions in oestrogen receptor‐positive, HER2‐negative early breast cancer. Evidence remains mixed regarding the survival benefit of adding chemotherapy to endocrine therapy (CET) in intermediate‐ and high‐risk RS groups. This meta‐analysis assessed overall mortality, breast cancer‐specific mortality (BCSM) and recurrence outcomes according to RS category.

**Methods:**

Medline, Embase, PubMed and Google Scholar were searched from 1 January to 20 June 2024 for studies reporting outcomes in ER+/HER2− patients treated with CET versus endocrine therapy (ET) alone, stratified by RS. Random‐effects modelling generated pooled risk ratios (RRs) with 95% confidence intervals (CIs). Heterogeneity was evaluated using *I* ^2^, and risk of bias was assessed with ROBINS‐I.

**Results:**

Fifteen studies comprising 630,741 patients were included. In the high‐risk group (RS > 25), CET significantly reduced 5‐year overall mortality compared with ET (5.9% vs. 7.9%; RR 0.57, 95% CI 0.45–0.72; *I* ^2^ = 91*%*). Node‐negative high‐risk patients also showed improved survival (RR 0.48, 95% CI 0.37–0.64; *I* ^2^ = 77*%*). BCSM was lower with CET in high‐risk patients (RR 0.81, 95% CI 0.67–0.97; *I* ^2^ = 0*%*). In the intermediate‐risk group (RS 11–25), CET did not significantly reduce overall mortality (RR 0.72, 95% CI 0.49–1.06; *I* ^2^ = 94*%*) or BCSM (RR 1.28, 95% CI 0.91–1.79; *I* ^2^ = 45*%*). Subgroup analysis of RS 16–25 showed lower overall mortality with CET (RR 0.48, 95% CI 0.42–0.55; *I* ^2^ = 0*%*), although BCSM was similar. Data for 5‐year recurrence outcomes were insufficient for pooled analysis.

**Conclusion:**

Chemotherapy provides a clear survival benefit in high‐risk RS groups, including node‐negative patients, whereas intermediate‐risk groups show limited benefit. These findings support selective use of CET guided by genomic risk stratification.

## 1. Introduction

Breast cancer remains one of the most common malignancies affecting women worldwide [1]. Among the various subtypes, oestrogen receptor‐positive (ER‐positive), human epidermal growth factor Receptor 2‐negative (HER2‐negative) breast cancer constitutes a significant proportion of cases. This subtype is characterised by the presence of oestrogen receptors on the surface of the cancer cells and the absence of overexpression or amplification of the HER2 gene, influencing the disease′s behaviour and response to treatment [2].

The prognosis and treatment strategies for ER‐positive, HER2‐negative breast cancer vary significantly depending on the risk of recurrence. The benefit of chemotherapy in patients with low risk of recurrence is low; hence, adjuvant chemotherapy is given to the moderate and high‐risk patients who have a combination of clinical and pathological risk factors, including tumour size, grade, lymph node involvement and genetic profiling tests such as Oncotype DX, Pan 50 and EndoPredict [3]. These tests provide a recurrence score (RS) or similar metrics that help stratify patients based on their risk of recurrence and guide treatment decisions for adjuvant chemotherapy [4].

Risk stratification using genetic profiling tests is crucial in guiding treatment decisions for ER‐positive, HER2‐negative breast cancer [5]. The Oncotype DX test provides a RS that categorises patients into low, medium and high‐risk groups [6]. This is one of the common tests used across North American and Europe. Patients with an RS below 18 are considered low risk and typically do not benefit from chemotherapy, relying instead on adjuvant endocrine therapy [7]. Those with an RS between 19 and 25 fall into the intermediate‐risk group, where the decision to use chemotherapy is more nuanced and individualised [8]. In these patients, adjuvant chemotherapy is generally advocated if they are premenopausal. High‐risk patients, with an RS of 25 or above, are typically strongly recommended to receive chemotherapy due to the significant reduction in recurrence risk and improved survival outcomes [4].

Oncotype DX, the most widely used test in the UK, offers a RS guiding chemotherapy decisions, and its utility has been further validated by the Trial Assigning Individualised Options for Treatment (TAILORx) trial, which specifically evaluated its effectiveness in a large cohort of patients [7]. This stratification framework enables clinicians to tailor treatment plans effectively, optimising therapeutic benefits while minimising unnecessary side effects. By leveraging these genetic tests, oncologists aim to make more informed decisions, ensuring that chemotherapy is appropriately utilised to enhance patient outcomes in those at medium to high risk of recurrence.

The TAILORx trial was a landmark study that evaluated the utility of the Oncotype DX test in guiding adjuvant chemotherapy decisions for women with ER‐positive, HER2‐negative breast cancer. This trial enrolled over 10,000 women and demonstrated that those with an intermediate RS of 11 to 25 could safely avoid chemotherapy without compromising their outcomes [7, 8]. However, in a subset of premenopausal patients, there was a benefit of chemotherapy if their RS was more than 19 [7]. These findings have significantly influenced clinical practice by providing strong evidence to support personalised treatment plans based on genetic profiling.

Similarly, the RESPONDER trial explored the benefits of adjuvant chemotherapy in patients with a higher risk of recurrence, emphasising the importance of individualised treatment approaches. The RESPONDER trial further validated the use of genetic tests such as Oncotype DX in identifying patients who would benefit most from chemotherapy, highlighting the balance between therapeutic efficacy and quality of life considerations [9].

The TAILORx and RESPONDER trials have shown a reduction in recurrence in patients with a RS of 19 and above who received chemotherapy. This led to increased use of Oncotype DX for most of the ER‐positive and HER2‐negative patients globally. In recent times, many national and population‐based registries have published data on the use of Oncotype DX and the long‐term survival of patients with ER‐positive and HER2‐negative breast cancer. Aggregation and assessment of this data provide a pragmatic overview of the benefit of chemotherapy in intermediate‐ and high‐risk patients categorised by Oncotype DX [9]. This study is aimed at conducting a systematic review and meta‐analysis of the population‐based studies to assess the benefit of chemotherapy on the long‐term outcome among ER positive and HER2 negative patients who were categorised as intermediate and high risk of recurrence based on their Oncotype DX score.

Preliminary analyses from this meta‐analysis were previously presented at conferences in poster form at the Association of Breast Surgery 2025 Conference [10], the Association of Surgeons in Training 2025 Conference [11] and the Shaukat Khanum Memorial Hospital 2025 Symposium [12].

## 2. Methods

### 2.1. Search Strategy

A comprehensive literature search was undertaken across Medline, Embase, PubMed and Google Scholar. The search covered the period from 1 January 2024 to 20 June 2024. Search terms included combinations of ‘early breast cancer’ or ‘breast cancer’ AND ‘oncotype DX’ or ‘oncotype’ or ‘gene profiling test’. Reference lists of included studies and relevant reviews were screened to identify any additional eligible publications. The methodological structure of this search was informed by our group′s previous meta‐analysis in breast surgical oncology, although the clinical question and search terms for the present study were entirely distinct [13].

### 2.2. Inclusion Criteria

Studies were selected according to predetermined PICO criteria.

### 2.3. Population/Participants

Patients with ER‐positive and HER2‐negative breast cancer who were categorised as intermediate risk and high risk of recurrence based on Oncotype DX gene profiling test. Population‐based studies with long‐term follow up were included in the review.

### 2.4. Intervention

The intervention of this study is endocrine therapy combined with any type of radiotherapy, adjuvant chemotherapy or surgical intervention.

### 2.5. Comparator

The comparator for this study is endocrine therapy alone versus combined endocrine therapy and chemotherapy.

### 2.6. Primary Outcomes

The primary outcomes are as follows:

1. 5‐year overall mortality.
2. 5‐year breast cancer‐specific mortality.

### 2.7. Secondary Outcomes

The secondary outcomes are as follows:

1. 5‐year local recurrence rate.
2. 5‐year distant recurrence rate.

## 3. Exclusion Criteria

Exclusion criteria included local single centre studies with mean follow up of less than 5 years.

### 3.1. Screening

Search results were imported into a reference manager and duplicates removed. Two reviewers independently screened titles and abstracts, followed by full‐text assessment using the predefined criteria. Any disagreements were resolved through discussion and final decision by a third reviewer. No machine‐assisted or automated tools were used at any stage.

### 3.2. Study Selection and Data Collection

**Figure 1:**
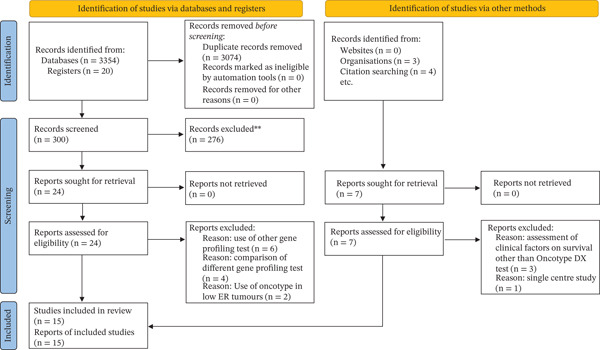
PRISMA of study selection. This figure illustrates the PRISMA flow of study selection. From 3374 records identified, 3074 were removed as duplicates, leaving 300 for screening. Following screening, 24 records from databases/registers and seven from other sources were assessed in full. Of these 31 full‐text reports, 16 were excluded with reasons, and 15 studies (15 reports) were included in the final review.

Eligible studies of any design were included, such as retrospective cohorts, prospective observational studies and database analyses. Data extraction was performed independently by two reviewers using a standardised proforma to capture study characteristics, patient demographics, RS groups, treatment arms and outcome measures. Extracted data were cross‐checked manually to ensure accuracy. No automation tools were used. A flow diagram of the study selection methods according to PRISMA can be found in Figure 1.

## 4. Statistical Analysis, Effect Measures and Risk of Bias Assessment

Meta‐analysis was conducted using a random‐effects model to account for between‐study variability. Effect sizes were calculated as risk ratios (RRs) with 95% confidence intervals, with *p* < 0.05 considered statistically significant. Heterogeneity was assessed using the *I* ^2^ statistic, with values above 50% indicating substantial heterogeneity.

Risk of bias for each included study was evaluated with the ROBINS‐I tool [17,18], assessing confounding, selection, classification of interventions, completeness of outcome data and reporting domains. Funnel plots were generated where appropriate to assess potential publication bias.

## 5. Results

### 5.1. Description of Studies

A total of 15 studies were analysed, involving 630,741 patients with hormone receptor‐positive, HER2‐negative breast cancer (Table 1). The included studies primarily focused on the prognostic and predictive value of RS testing in patients receiving adjuvant chemotherapy or endocrine therapy. Patient cohorts ranged from node‐negative to node‐positive, with risk groups classified based on RS cutoffs commonly utilised in the TAILORx trial (RS 11‐25 intermediate‐risk, RS > 25 high risk). Across studies, chemotherapy was generally associated with improved OS and BCSM in patients with high RS, especially in premenopausal patients and those under 50 years of age. However, in low to intermediate‐risk groups, the benefit of chemotherapy was less consistent, with several studies showing no significant difference in survival outcomes compared with endocrine therapy alone. Study designs were retrospective cohort analyses, examining outcomes such as 5‐year mortality rate, 10‐year distant recurrence rates and secondary invasive breast events (SIBE) across varying clinical parameters, including patient age, tumour stage and nodal status. The ROBINS‐I tool assessed risk of bias, identifying that 13 studies were of moderate risk and four of low risk, reflecting robust methodologies in capturing the long‐term benefits of adjuvant chemotherapy in distinct patient subgroups (Table 1).

**Table 1:** Study descriptive results and demographics of studies included in analysis.

Author and year of publication	Total patients	Database used	Year of data collection	Lobular ca proportion(*n*[%])	Proportion of G1/2 (%)	Proportion T1/2 tumours	Node status (positive or negative)	Menopausal status (pre or post menopausal only)	RS score cut‐off	Outcomes reported	RoB score
Kizy et al. [14]	7316	SEER	2004–2013	100	93	97	Both	Both	11–25 and > 25	BCSS	Moderate
Ma et al. [15]	5054	SEEER	2010–2013	20.8	67.2	100	Both	Both	> 25	OS	Moderate
Choi et al. [16]	89,402	SEER	2004–2015	10.1	72.8	100	Negative	Pre	11–25 and > 25	BCSS	Low
Stemmer [17]	1801	CHS	N/A	11.8	64.7	N/A	Negative	Both	N/A	DRFS	Moderate
Stemmer [18]	1365	CHS	2006–2009	11.7	64.7	N/A	Negative	Both	18–25 and > 25	DRFS and OS	Moderate
Yang 2020 [19]	3754	SEER	2004–2013	N/A	88.7	100	Negative	Both	25–30	BCSS	Moderate
Ding [20]	7965	SEER	2010–2015	12.7	11.0	96.7	Positive	Both	15–25	OS	Low
Iorgulescu [21]	21,144	NCDB	2010–2015	3.9	0 (all G3)	100	Both	Both	> 30	OS	Low
Park [22]	29,137	SEER	2004–2015	29.2	64.7	98.4	Negative	Both	25–30	BCSS and OS	Moderate
Chen [23]	21,991	NCDB	2006–2012	N/A	82	100	Negative	Both	11–15	OS	Moderate
Weiser [24]	115,833	NCDB	2004–2018	14	80.1	99	Both	Both	> 25	OS	Moderate
Nash [22]	15,422	NCDB	2010–2017	N/A	75.7	N/A	Both	Pre	> 25	OS	Low
Kumar [25]	15,792	NCDB	2010‐2018	9.7	84.8	98.9	Negative	Pre	16–25	OS	Moderate
Stabillini 2022 [14]	16,745	NCDB	N/A	N/A	N/A	N/A	Negative	Post	> 25	OS	Moderate
Roy [26]	553,497	NCDB	2010–2017	12.5	82.4	99.6	Both	Both	16–25 and > 25	OS	Moderate

*Note:* This table illustrates an overview of the descriptive results and demographic details of studies included in the analysis. Information includes the authors, publication year, total patients, database sources and year of data collection. Key characteristics described including lobular cancer proportions, grading (G1/2), tumour size (T1/2), node status, menopausal status, RS cut‐offs, outcomes reported and risk of bias (RoB) scores. This demonstrates an overall comparative view of study populations and methodologies of the included studies.

Abbreviations: BCSS, breast cancer‐specific survival; DRFS, distant recurrence‐free survival; OS, overall survival.

Kumar et al. [25]—A retrospective analysis on 15,972 premenopausal, hormone receptor positive breast cancer patients comparing survival of patients who had adjuvant chemotherapy or none. Patients who received adjuvant chemotherapy were more likely to have certain associated factors, such as higher T stage, poor and moderately differentiated tumours, age over 40 years, care at an academic centre, Caucasian race, patients undergoing mastectomy, regional lymph node surgery and radiation therapy. The analysis showed an overall survival benefit for adjuvant chemotherapy use in ER‐positive, N0 premenopausal BC patients, age less than 50 years, with an intermediate RS score, particularly 21–25.

Stemmer et al. [18]—A retrospective analysis on 1365 node negative ER‐positive HER2‐negative breast cancer patients who underwent RS testing. Authors split the cohort based on RS scores and compared BCSM and 10‐year distant recurrence rates for patients who had adjuvant chemotherapy versus endocrine therapy alone. It was found that in patients with RS 11–25 there was no difference in 10‐year distant survival or BCSM rates between CT and ET patients.

Ding et al. [20]—A retrospective cohort study on 7965 lymph node positive ER‐positive HER2‐negative RS 14‐25 patients. There were two groups: 5774 (72.5%) patients receiving no chemotherapy and 2191 (27.5%) who had chemotherapy. In those who had RS 14–25 invasive ductal carcinoma and 2–3 positive lymph nodes, there was a significant 5‐year overall survival benefit. The weighted 5‐year overall survival rates were higher for patients in the chemotherapy group compared with those in the nonchemotherapy group, and the same was demonstrated in high‐risk patients, with a significant absolute difference of 1.8%.

Chen et al. [23]—A retrospective cohort study on 21,991 ER‐positive HER2‐negative patients who had RS 11‐25 and stage T2N0M0. Patient were divided into chemotherapy and nonchemotherapy groups, and analysed which variables affected overall survival in each group. Authors report younger patients were more likely to have received chemotherapy and, overall, worse overall survival was demonstrated in those with Black race, treatment at a community programme, Medicaid, high‐grade tumours, pT2 versus pT1c, higher Charlson–Deyo score, and no radiotherapy. In terms of the benefit of chemotherapy, it was found that there was no significant difference in overall survival between those who received chemotherapy and those who did not (97.4% vs. 97.8%).

Choi et al. [16]—A retrospective statistical analysis of 89,402 patients with hormone receptor positive, node negative, early‐stage breast cancer, who had a calculated RS score. Cohorts were split into three groups according to RS score: RS < 11, low risk; RS 11–25, intermediate risk; RS > 25, high risk. In the high‐risk group, chemotherapy was significantly associated with a reduced risk of breast cancer related mortality, but in the low and intermediate RS groups, there were no significant differences in BCSM between patients who received chemotherapy and those who did not.

Kizy et al. [14]—A retrospective analysis of 7316 patients aged 18–74 years, with ER positive ILC and an available RS. They split patients into risk groups based on the TAILORx trial and traditional cut‐offs and assessed the 5‐year breast cancer‐specific survival between chemotherapy and nonchemotherapy patients. A total of 21%, 71% and 8% of patients were in low, intermediate and high‐risk groups, respectively. Results showed that in the high‐risk group, the RS predicted a lower 5‐year BCSS, and that adjuvant chemotherapy did not add survival benefit to the intermediate or high‐risk cohorts.

Stabellini et al. [24]—A retrospective statistical analysis of 16,745 women who were aged > 50 (postmenopausal), had received surgery and endocrine therapy, and had ER+/HER2− pT1‐2N0M0 breast cancer with RS > 26. This paper compared 5‐year OS rates between ET and CET patients and revealed that 5‐year OS was higher in CET patients. Authors also found chemotherapy use had increased over time.

Iorgulescu et al. [21]—A retrospective statistical analysis of 30,864 women with pT1c to pT2, pN0 to pN1, Grade 3 ER‐positive, HER2–negative invasive breast carcinoma from 2010 to 2015. This study compared the 5‐year overall survival rate for patients between RS groups and found that patients in the high‐risk group (RS > 31) benefitted significantly from chemotherapy compared with intermediate and low risk groups.

Yang et al. [19]—A retrospective analysis of 3754 early‐stage breast cancer patients, who had RSs calculated. The specific aim was to assess benefits of chemotherapy among patients with RS 26–30. It was found that patients with higher tumour grade, larger tumour size and younger patients were more likely to receive chemotherapy. Furthermore, it was shown that receipt of chemotherapy was independently associated with better breast cancer specific survival in patients with RS 26–30.

Park et al. [22]—A retrospective study of 29,137 node‐negative, hormone receptor‐positive and HER2‐negative breast cancer patients with RS 18–30, spanning over 11 years. Of the 21% of patients who had RS 26–30 and were 70 years of age and below, chemotherapy administration was linked to a 32% lower breast cancer‐specific mortality, and a 42% lower overall mortality. These survival benefits were greater in those who were younger or who had high‐grade tumours.

Weiser et al. [27]—A retrospective analysis of 115,833 hormone receptor positive, HER2 negative breast cancer patients with diagnoses of ILC and IDC and an available RS. Authors aimed to assess the prognostic and predictive value of using RS in ILC patients. In their cohort, ILC patients with high RS and N0 or N1 disease had 10% less chemotherapy than their counterparts with IDC. An absolute overall survival advantage was associated with chemotherapy receipt in ILC patients with high RS and who had N0 or N1 disease. Overall, RS had statistically significant prognostic value for ILC patients.

Ma et al. [15]—A retrospective analysis on 5054 breast cancer patients diagnosed between 2010 and 2013 with hormone receptor‐positive, HER2‐negative and T1‐2N0 breast cancer with a RS ≥ 26. RS subgroups were divided into RS 26–30 and RS > 30 and found that in the former group, adjuvant chemotherapy added a statistically significant overall survival benefit. It was also found that those with RS > 30 who underwent chemotherapy had increased mortality compared with their counterparts with RS 26–30.

Stemmer et al. [17]—A retrospective cohort analysis of 1801 patients with N0 ER + HER2 − negative breast cancer and RS testing available. Authors utilised the same RS categories as the TAILORx trial, with a particular interest in patients with RS ≥ 11 due to lacking data on clinical outcomes at the time. Among the subgroups, chemotherapy use showed no statistically significant difference in outcomes (even after propensity score adjustment). Furthermore, patients not treated with chemotherapy with RS 11–25 had very low distant recurrence rates, suggesting that chemotherapy may not have had a clinically meaningful benefit.

Nash et al. [28]—A retrospective statistical analysis of 15,422 patients aged 40–50, diagnosed with HR‐positive, HER2‐negative breast cancer between 2010 and 2017. They grouped patients by age, nodal status, RS and receipt of chemotherapy. They found that of the 45.3% of patients who received chemotherapy, there was a significant overall survival benefit in those with pN1 disease and RS of 31–50.

Roy et al. [26]—A retrospective cohort study of 553,497 patients diagnosed with resectable HER2‐low or HER2‐zero, early‐stage breast cancer. They aimed to assess the prognostic advantage of RS testing for HER2‐low and HER2‐zero cancers. Adjuvant and neoadjuvant chemotherapy use showed an overall survival advantage in HER2‐low patients regardless of hormonal receptor status, even after adjusting for RS. This was similarly observed in hormone receptor positive patients with high RS (26–100). They also concluded that resectable HER2‐low breast cancer had a better prognosis than HER2‐zero.

### 5.2. Primary Outcomes

#### 5.2.1. High‐Risk Group (RS > 25)

**Figure 2:**
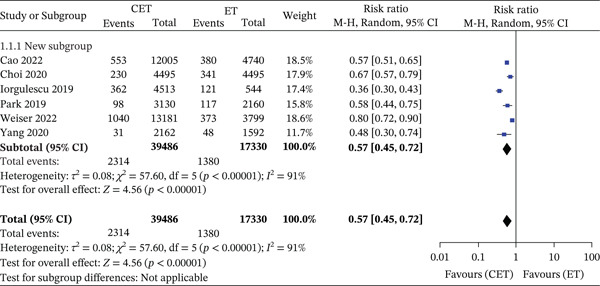
Forest plot for 5‐year overall mortality—high‐risk group. Forest plot displaying the 5‐year overall mortality risk ratio for ‘high‐risk group’ patients across multiple studies, comparing CET (chemotherapy and endotherapy) with ET (endotherapy). Heterogeneity is significant (*I* ^2^ = 91*%*), and the overall risk ratio favours CET (RR = 0.57 [0.45, 0.72], *p* < 0.00001).

Across six studies, 5‐year overall mortality was significantly lower in patients receiving CET compared with ET (2314/39,486 [5.9%] vs. 1380/17,330 [7.9%]; RR 0.57, 95% CI 0.45–0.72; *p* < 0.001), with substantial heterogeneity (*I* ^2^ = 91*%*) (Figure 2).

**Figure 3:**
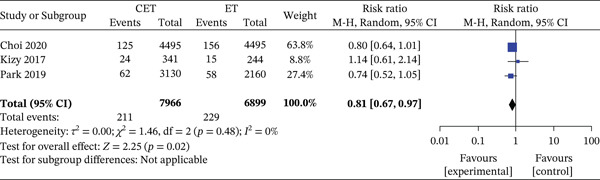
Forest plot for breast cancer‐specific mortality in high‐risk group. Forest plot comparing breast cancer‐specific mortality between the CET (chemotherapy and endotherapy) and ET (endotherapy) groups in high‐risk patients across three studies. The number of events (CET: 211/7966, ET: 229/6899) and the pooled risk ratio (RR = 0.81 [0.67, 0.97], *p* = 0.02) indicate a statistically significant reduction in mortality with CET. The analysis shows no heterogeneity among studies (*I* ^2^ = 0*%*), suggesting consistency in results across studies.

In node‐negative high‐risk patients, CET was likewise associated with improved survival (901/18,039 [4.9%] vs. 522/6791 [7.7%]; RR 0.48, 95% CI 0.37–0.64; *p* < 0.001; *I* ^2^ = 77*%*). High‐risk patients also experienced a reduction in breast cancer‐specific mortality with CET (211/7966 [2.6%] vs. 229/6899 [3.3%]; RR 0.81, 95% CI 0.67–0.97; *p* = 0.02; *I* ^2^ = 0*%*) (Figure 3).

#### 5.2.2. Intermediate Group (RS 11–25)

**Figure 4:**
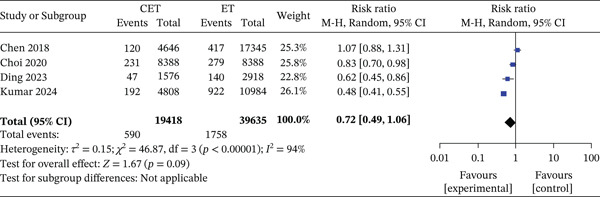
Forest plot for 5‐year overall mortality—intermediate‐risk group. Forest plot comparing 5‐year overall mortality between the CET (chemotherapy and endotherapy) and ET (endotherapy) groups in intermediate‐risk patients (RS 11–25) across four studies. The number of events (CET: 590/19418 [3.1%], ET: 1758/39635 [1.5%]) and the pooled risk ratio (RR = 0.72 [0.49, 1.06], *p* = 0.09) indicate no statistically significant difference in mortality between CET and ET groups. High heterogeneity is observed among studies (*I* ^2^ = 94*%*).

Four studies reported overall mortality, showing no significant difference between CET and ET (590/19,418 [3.1%] vs. 1758/39,635 [1.5%]; RR 0.72, 95% CI 0.49–1.06; *p* = 0.09; *I* ^2^ = 94*%*). BCSM likewise showed no significant difference (151/11,031 [1.4%] vs. 198/16,276 [1.2%]; RR 1.28, 95% CI 0.91–1.79; *p* = 0.15; *I* ^2^ = 45*%*) (Figure 4).

In this higher‐intermediate subgroup, CET was associated with lower overall mortality (224/6061 [3.7%] vs. 1086/14,248 [7.6%]; RR 0.48, 95% CI 0.42–0.55; *p* < 0.001; *I* ^2^ = 0*%*). BCSM remained comparable between groups (34/1333 [2.6%] vs. 142/4037 [3.5%]; RR 0.81, 95% CI 0.35–1.90; *p* = 0.63; *I* ^2^ = 40*%*).

#### 5.2.3. 5‐Year Local Recurrence and Distant Recurrence Rate

There was not enough data to conduct meta‐analysis on 5‐year recurrence rate. Stemmer et al. (27) mentioned the overall recurrence rate in medium risk group to be *n* = 6/80 (7.5%) in CET group versus *n* = 34/773 (4.4%) in ET group.

## 6. Discussion

The results of this study demonstrate that the reduction in 5‐year overall and breast cancer‐specific mortality with chemotherapy was 2.0% and 0.7%, respectively. However, there was significant heterogeneity between the studies for the overall mortality rate. There was no difference in overall and breast cancer‐specific mortality in the chemotherapy group versus those with endotherapy alone in the intermediate risk group. However, there was a reduction in the overall mortality rate by 3.9% with the use of chemotherapy in the subgroup of the intermediate group with the RS score between 16 and 25.

For intermediate‐risk patients, the benefit of CET is less clear. The overall mortality did not show a significant difference between CET and ET groups, and breast cancer‐specific mortality was higher in the CET group, although this result was not statistically significant. The population‐based studies have assessed long‐term outcomes with the RS cut‐off 11–25. The subgroup analysis in the clinical trial showed the benefit of chemotherapy patients for those with RS cut‐off above 16, and in particular above 20, in the intermediate risk group. We were not able to conduct an analysis on this RS cut‐off as done in the clinical trial for fair comparison. The population‐based studies that included patients with RS cut‐off above 16–25, reduction in overall mortality.

The role of chemotherapy in the high‐risk group is evident, providing a substantial reduction in mortality rates. This aligns with current guidelines recommending chemotherapy for patients with a high RS to improve survival outcomes. The heterogeneity in the high‐risk group suggests that although the overall trend supports chemotherapy, individual patient characteristics and study differences must be considered. In the intermediate‐risk group, our results align with findings from the TAILORx trial, which suggested that patients with an RS of 11–25 might not benefit significantly from chemotherapy. Our subgroup analysis for patients with an RS of 16–25 showed a lower overall mortality with CET, yet no significant difference in breast cancer‐specific mortality. This underscores the complexity of treatment decisions in this group and suggests that chemotherapy should be considered on a case‐by‐case basis, potentially guided by additional clinical and molecular factors. The RESPONDER trial also supports our findings for high‐risk patients, reinforcing the use of genetic tests like Oncotype DX to guide chemotherapy decisions. The clear benefit of chemotherapy in reducing mortality for high‐risk patients corroborates our analysis and highlights the effectiveness of genetic profiling in stratifying treatment approaches. However, the variability in outcomes for intermediate‐risk patients observed in our study reflects the ongoing need for individualised treatment strategies and further research in this area.

There are other gene profiling tests used in different breast units. The Pan 50 and EndoPredict tests, alongside others such as Prosigna, Epclin and MammaPrint, offer similar stratifications by identifying low, intermediate and high‐risk categories based on gene expression profiles. Prosigna (based on the PAM50 gene signature) provides a risk of RS, helping to determine the necessity of chemotherapy in postmenopausal women with hormone receptor‐positive early‐stage breast cancer [29]. Epclin, part of the EndoPredict test, combines gene expression data with clinical factors to refine risk assessment [30]. The MammaPrint test uses a 70‐gene signature to classify patients into high or low risk of recurrence [31]. In the preliminary search, we did not find large population studies that have assessed long‐term mortality outcome in the patients stratified as medium and high‐risk using tests other than Oncotype DX. Some of these gene‐profiling tests are widely used in some regions and more long‐term survival outcome data is vital.

There were certain limitations in the study. Despite best efforts, most studies had narrow inclusion criteria for the long‐term assessment. Some studies focused on intermediate risk, whereas others assessed long‐term outcome in high‐risk patients. The cut‐off for RS was also variable across the studies. This made the aggregation of data difficult for the intermediate risk group. Ideally, we would like to analyse the long‐term outcome in a subgroup of intermediate risk with RS between 16 and 25, which is more pragmatic and used for clinical management of the patients. This was not possible in most studies as the intermediate risk group RS score was based on previous trials that included patients with RS between 11 and 25. Similarly, some studies assessing high‐risk groups have used RS score cut‐off values of more than 30, rather than 25 that is more clinically relevant. We also planned to include other genetic profiling tests; however, there is limited long‐term population‐based data on their survival and recurrence, so our research team decided to focus the review on Oncotype DX.

Most of the data on Oncotype DX has come from cancer registries in United States. As shown in Table 1, there is also overlap of the years in studies that have retrieved data from the same national registry. However, the inclusion criteria for these studies were different. Hence, we included all these studies. Oncotype DX test is primarily used to categorise patients based on their risk of recurrence. The clinical trials conducted have recurrence rates as their primary objective. However, in this review, the main objective was to assess long‐term mortality rate. Some studies have shown that those with a high‐RS score have poor survival, but it is also important to assess if chemotherapy given to these high‐risk patients has any impact on their survival. The study′s second objective was to assess recurrence rate in the patients; however, most population‐based studies did not mention long‐term recurrence rate.

Future research should focus on refining risk stratification methods to better identify intermediate‐risk patients who would benefit most from chemotherapy. Incorporating more extensive molecular profiling and exploring new biomarkers could enhance predictive accuracy. Additionally, prospective trials that include QoL assessments alongside survival outcomes are needed to better understand the trade‐offs involved in chemotherapy decisions as this is an important patient factor to consider in complex decision‐making [32]. Personalised treatment plans should consider both clinical efficacy and patient well‐being, ensuring that therapy is tailored to individual needs and preferences.

## 7. Conclusion

Adjuvant chemotherapy combined with endocrine therapy demonstrated a clear survival advantage in patients with high genomic risk disease (RS greater than 25), reflected by reduced 5 year overall and breast cancer‐specific mortality. Substantial heterogeneity across studies evaluating overall mortality suggests underlying variation in population characteristics and treatment patterns, yet the overall direction of effect consistently favoured chemotherapy.

In contrast, patients categorised within the intermediate risk range did not experience a meaningful reduction in either overall mortality or breast cancer‐specific mortality when chemotherapy was added to endocrine therapy. Although certain subgroups, particularly those with RS between 16 and 25, showed signals of improved overall survival, this was not accompanied by a corresponding reduction in breast cancer‐specific mortality, highlighting the complexity of treatment decision‐making in this cohort.

Together, these findings reinforce the role of genomic profiling in identifying individuals most likely to benefit from chemotherapy. They also underscore the need for more granular risk evaluation within the intermediate risk category, integrating molecular features, clinical factors and future prospective evidence to enable more personalised and clinically balanced treatment recommendations.

## Funding

This research received no external funding. Open access funding provided by Imperial College London.

## Conflicts of Interest

The authors declare no conflicts of interest.

## Data Availability

The data that support the findings of this study are available in References 12–14, 16, 18–25, 27, 33, 34 at https://www.doi.org/. These data were derived from the following resources available in the public domain: [25] Kumar et al. 2024, https://ascopubs.org/doi/10.1200/PO.23.00390; [18] Stemmer et al. 2019, https://www.nature.com/articles/s41523-019-0137-3; [20] Ding et al.2023, https://linkinghub.elsevier.com/retrieve/pii/S1526820923001908; [23] Chen et al. 2018, https://breast-cancer-research.biomedcentral.com/articles/10.1186/s13058-018-0957-3; [16] Choi et al. 2020, https://www.mdpi.com/2072-6694/12/7/1829; [14] Kizy et al. 2017, https://link.springer.com/article/10.1007/s10549-017-4355-9[24] Stabellini et al. 2023, https://onlinelibrary.wiley.com/doi/10.1002/cam4.6584; [21] Iorgulescu et al. 2019, https://ascopubs.org/doi/10.1200/PO.19.00029; [19] Yang et al. 2021, https://www.tandfonline.com/doi/full/10.2217/fon-2020-1315; [22] Park et al. 2019, https://breast-cancer-research.biomedcentral.com/articles/10.1186/s13058-019-1190-4; [27] Weiser et al. 2022, https://acsjournals.onlinelibrary.wiley.com/doi/10.1002/cncr.34127; [15] Ma et al. 2021, https://onlinelibrary.wiley.com/doi/epdf/10.1111/tbj.14130; [17] Stemer et al. 2017, https://www.nature.com/articles/s41523-017-0033-7; [28] Nash et al. 2023, https://link.springer.com/article/10.1245/s10434-022-12699-3; [26] Roy et al. 2023, https://www.mdpi.com/2072-6694/15/17/4264.
